# Primary seminal vesicle diffuse large B-cell lymphoma: a case report and review of the literatures

**DOI:** 10.3389/fimmu.2024.1461090

**Published:** 2024-10-30

**Authors:** Youli Li, Sufen Cao, Fangfang Lv, Guang-Liang Chen

**Affiliations:** ^1^ Department of Medical Oncology, Fudan University Shanghai Cancer Center, Shanghai, China; ^2^ Department of Oncology, Shanghai Medical College Fudan University, Shanghai, China; ^3^ Department of Medical Oncology, Fudan University Shanghai Cancer Center Xiamen Hospital, Xiamen, China; ^4^ Department of Nursing, Fudan University Shanghai Cancer Center, Shanghai, China

**Keywords:** primary seminal vesicle lymphomas, diffuse large B-cell lymphoma, prostate cancer, genetic mutations, case report

## Abstract

Primary seminal vesicle lymphoma is a remarkably rare condition, predominantly manifesting as diffuse large B-cell lymphoma. Due to its rarity and nonspecific clinical presentations, it is often misdiagnosed or overlooked. Here, we report a case of a 68-year-old male diagnosed with primary seminal vesicle lymphoma, coinciding with prostate cancer. The diagnosis followed initial findings of elevated prostate-specific antigen levels and abnormal magnetic resonance imaging of the prostate and left seminal vesicle. Suspicion of prostate cancer led to a radical resection of both the prostate and seminal vesicle. Subsequent pathological examination and next-generation sequencing post-surgery confirmed the diagnosis of primary seminal vesicle diffuse large B-cell lymphoma, characterized by CD79B mutation type (MCD type). The patient was treated with six cycles of the R-CHOP regimen (rituximab, cyclophosphamide, vincristine, doxorubicin, prednisone), achieving complete metabolic remission as confirmed by positron emission tomography-computed tomography. Fifteen months post-treatment, the patient’s condition remains favorable. Through our literature review of additional six cases of primary seminal vesicle lymphoma, we aim to elucidate the typical clinical presentations, imaging features, pathological characteristics, genetic mutations, and therapeutic strategies, aiming to contribute to better detection and management of this rare malignancy. This case underscores the diagnostic challenges and emphasizes the necessity for heightened clinical suspicion and definitive pathological examination in the management of primary seminal vesicle lymphoma.

## Introduction

Primary extranodal lymphomas account for approximately one-third of all non-Hodgkin lymphomas (1). These malignancies may originate from almost any organ (2), but they most frequently involve the gastrointestinal tract, head and neck regions, orbit, central nervous system, lung, bone, and skin (1). Lymphomas within the genitourinary tract are exceedingly rare, and seminal vesicle involvement in primary extranodal lymphoma is rarer than 5% (3, 4).

Primary seminal vesicle lymphomas are defined as those localized in the seminal vesicles only, with no evidence of extranodal disease in any other part of the body or invasion from adjacent organs (5, 6). Primary diffuse large B-cell lymphoma (DLBCL) of the seminal vesicles is even rarer, and patients present with non-specific clinical symptoms which may initially be misdiagnosed as prostate cancer, or recognized but assumed to represent involvement of the seminal vesicle by prostate carcinoma (5). This misdiagnosis, then, can simply delay the appropriate therapeutic approach.

To date, literature reports are scant, with only six documented cases of primary lymphoma of the seminal vesicles (7–12), including four instances of DLBCL and two of Burkitt lymphoma. This rarity underscores the importance of raising awareness and understanding this disorder further for better clinical diagnosis and patient care.

Herein is reported one rare case of primary seminal vesicle DLBCL featuring CD79B^mut^ subtype (MCD type) in a 68-year-old man, who was concurrently diagnosed with prostate cancer. This case significantly contributes to the limited literature by providing an extensive review of the clinical presentations, imaging modalities, pathologic assessments, genetic findings, treatment approaches, and prognostic evaluations. Additionally, it underscores the clinical challenges and complexities associated with diagnosing and managing such uncommon cases.

## Case presentation

**Figure 1:**
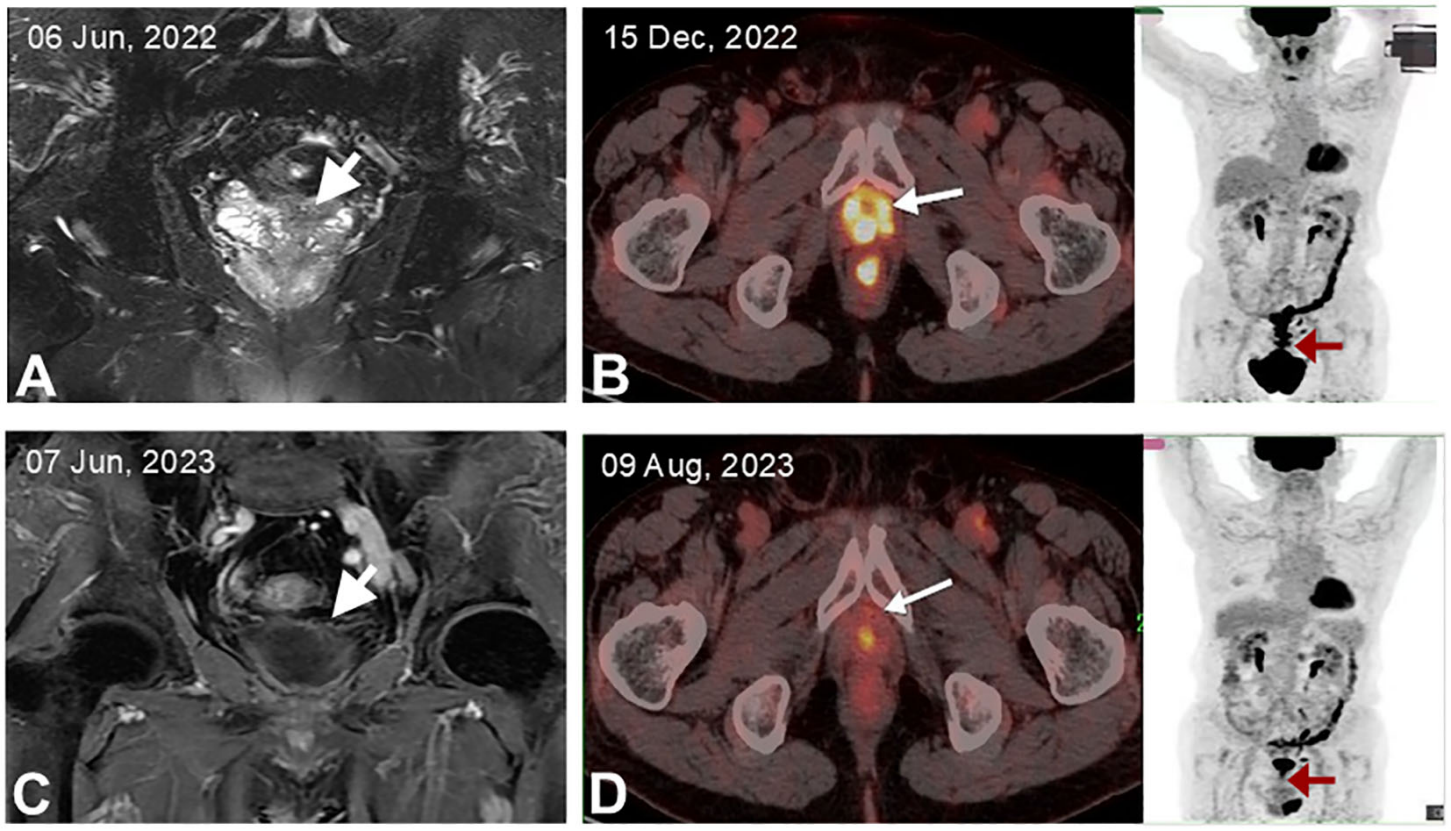
MRI and FDG-PET/CT imaging of the patient. **(A)** Initial MRI showing abnormal signals in the prostate and left seminal vesicle. **(B)** MRI after six cycles of R-CHOP treatment, indicating resolution of abnormal masses in the prostate and left seminal vesicle. **(C)** PET/CT post-radical prostatectomy, highlighting a maximum standardized uptake value (SUVmax) of 6.0 in the prostate fossa, with the arrow marking the specific loci. **(D)** PET/CT after completing six cycles of R-CHOP treatment, demonstrating a return to normal metabolic activity in the prostate fossa.

**Figure 2:**
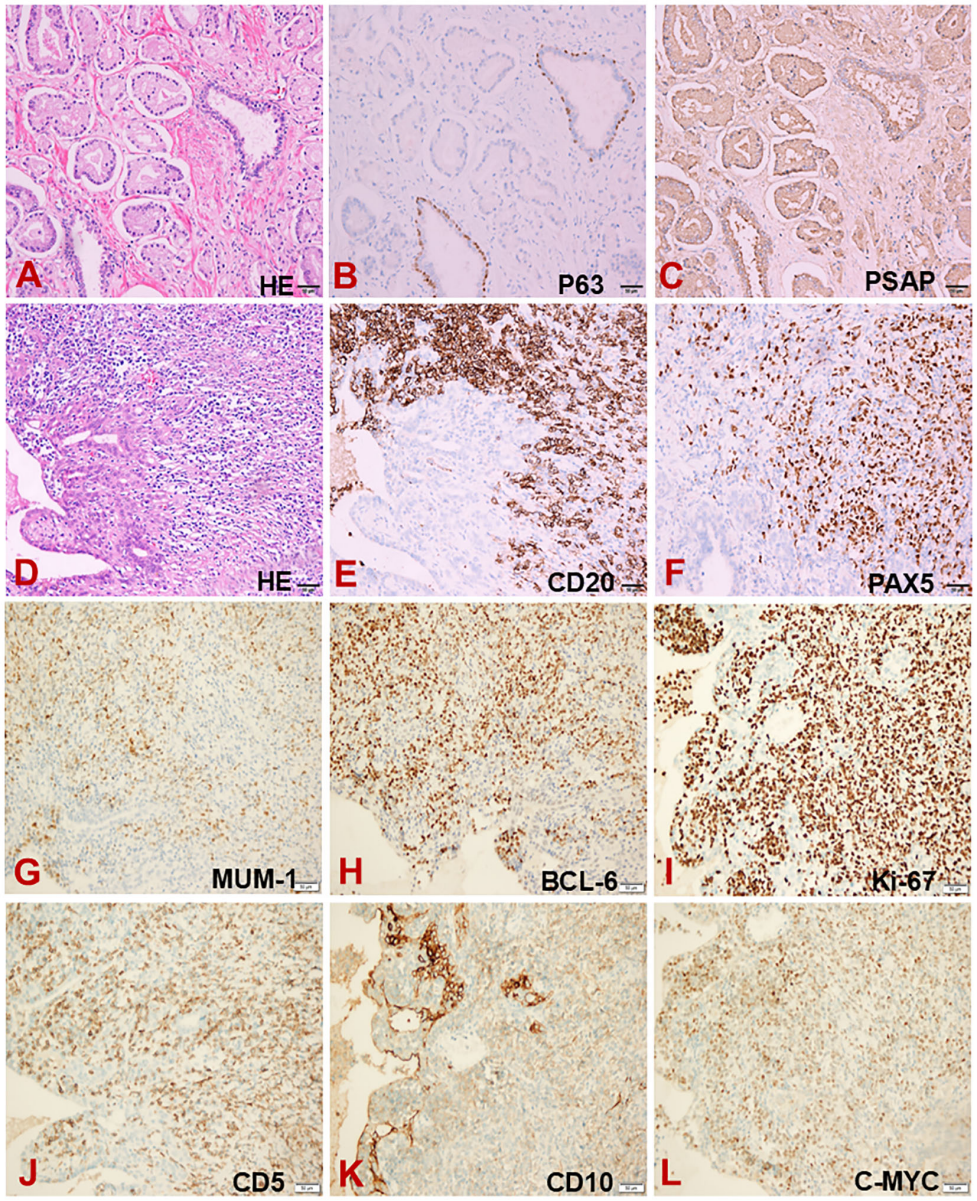
Pathological analysis of the prostate and seminal vesicles. **(A)** Hematoxylin and eosin staining of the prostate. **(B, C)** Immunohistochemical staining of the prostate for P63 **(B)** and PSAP **(C)**. **(D)** Hematoxylin and eosin staining of the seminal vesicles. **(E–L)** Immunohistochemical staining of the seminal vesicles for CD20 **(E)**, PAX5 **(F)**, MUM-1 **(G)**, BCL-6 **(H)**, Ki-67 **(I)**, CD5 **(J)**, CD10 **(K)** and C-MYC **(L)**.

**Figure 3:**
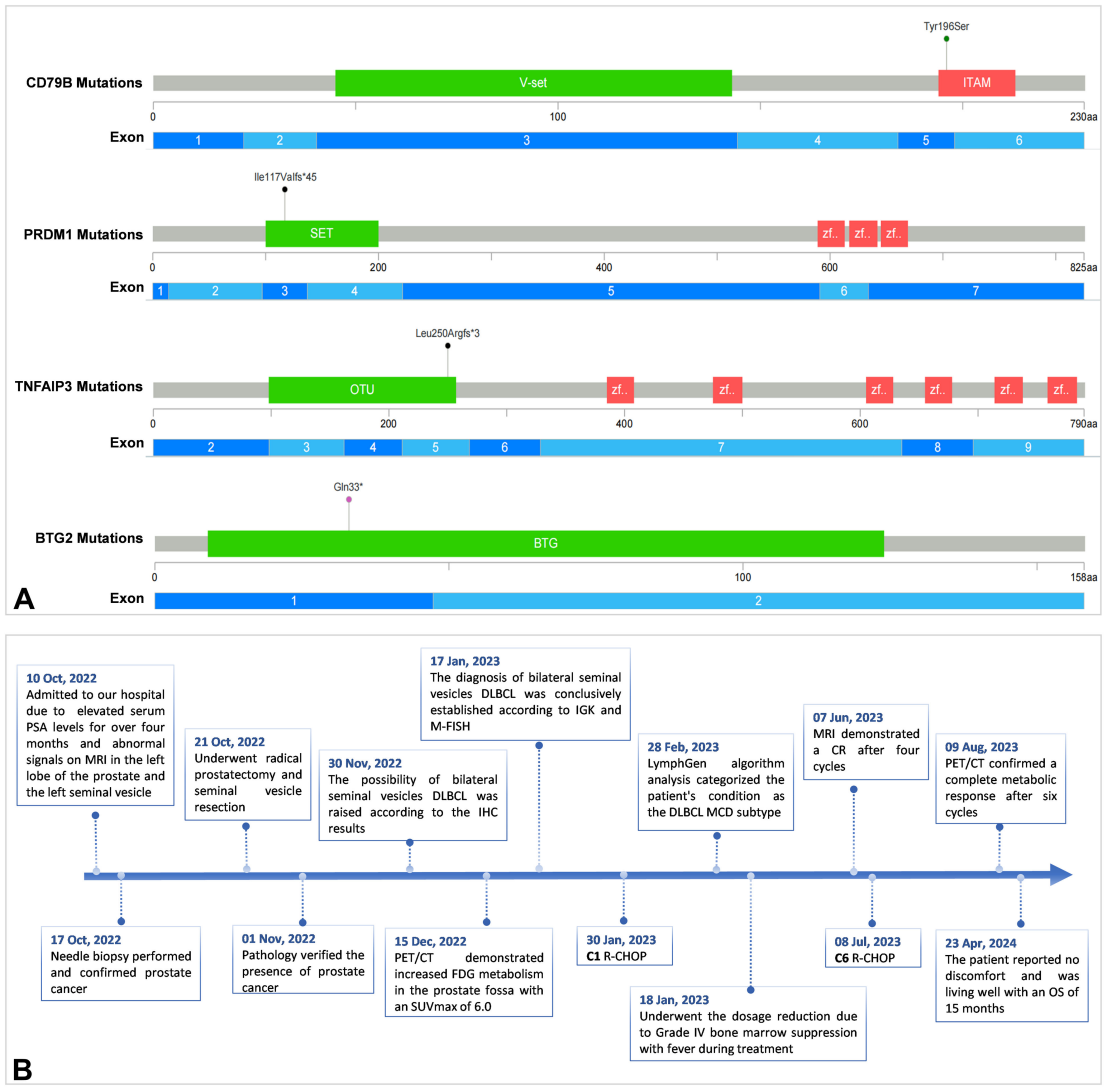
Pathogenic or likely pathogenic multiple somatic mutations of the patient **(A)** and the timeline of the patient care **(B)**.

A 68-year-old man with a longstanding history of hypertension and chronic nephritis was admitted to our hospital on Oct. 10, 2022, due to persistently elevated serum prostate-specific antigen levels noted for over four months. The physical examination of the patient revealed no abnormalities, and superficial lymph nodes were not noticeably enlarged. Pre-admission magnetic resonance imaging (MRI) scans demonstrated abnormal signals in the left lobe of the prostate and the left seminal vesicle (**Figure 1A**). A subsequent needle biopsy performed on Oct. 17, 2022, confirmed prostate cancer with a Gleason score of 3 + 4. The patient underwent radical prostatectomy and seminal vesicle resection on Oct. 21, 2022. The postoperative pathological examination, completed on Nov. 1, 2022, verified the presence of prostate cancer (**Figures 2A–C**). Notably, the pathology report dated Nov. 30, 2022, revealed atypical proliferation of lymphoid tissue in the bilateral seminal vesicles, primarily characterized by B-cell proliferation. The diagnosis of B-cell lymphocytic lymphoma should include considerations of DLBCL, high-grade/transformed follicular lymphoma, or Burkitt lymphoma, based on the immunohistochemical profile showing positive staining for CD20, CD10, Bcl-6, CD5, PAX5, C-MYC (50%), MUM1, and Ki-67 (80%), along with negative staining for CD3 and Bcl-2 (**Figures 2D–L**). The patient underwent further investigation with a whole-body postoperative positron emission tomography-computed tomography (PET/CT) scan on Dec. 15, 2022, which demonstrated increased fluorodeoxyglucose (FDG) metabolism solely in the prostate fossa, with an SUVmax of 6.0, and no evidence of tumor involvement elsewhere (**Figure 1C**). The baseline bone marrow aspiration biopsy confirmed the absence of lymphoma invasion. Additionally, PET/CT scans prior to chemotherapy indicated no tumor involvement in the nervous system. Clonal rearrangement of the immunoglobulin kappa (IGK) gene was confirmed, while multicolor fluorescence *in situ* hybridization (M-FISH) tests found no translocations involving Bcl-2, Bcl-6, and C-myc genes. Integrating these findings, the diagnosis was considered more likely to be primary bilateral seminal vesicle DLBCL on Jan. 17, 2023, but some involvement of prostate cancer in the prostate fossa cannot be excluded completely due to the concurrent presence of prostate cancer. On Feb. 28, 2023, genetic analysis of DNA extracted from paraffin-embedded tissue samples from the patient revealed multiple somatic mutations classified as pathogenic or likely pathogenic (**Figure 3A**). These included: a missense mutation in exon 5 of CD79B (NM_000626.4) (c.587A>C, p.Tyr196Ser), a frameshift mutation in exon 3 of PRDM1 (NM_001198.4) (c.348_351del, p.Ile117Valfs*45), a frameshift mutation in exon 5 of TNFAIP3 (NM_001270507.2) (c.749_750del, p.Leu250Argfs*3), and a nonsense mutation in exon 1 of BTG2 (NM_006763.3) (c.97C>T, p.Gln33*), with allele frequencies of 23.65%, 11.89%, 9.36%, and 5.62% respectively. Additionally, LymphGen algorithm analysis (13) categorized the patient’s condition as the DLBCL MCD subtype.

The patient underwent six cycles of the R-CHOP regimen (rituximab, cyclophosphamide, vincristine, doxorubicin, prednisone). Unfortunately, he showed significant intolerance to the immunochemotherapy, developing grade IV leukopenia (0.7 x 10^9/L) and grade IV granulocyte deficiency (0.25 x 10^9/L) following the first chemotherapy cycle. This adverse reaction necessitated dose reductions in subsequent cycles. MRI demonstrated a complete response after four cycles (**Figure 1B**), and PET/CT confirmed a complete metabolic response after six cycles with no tumor involvement in the nervous system (**Figure 1D**). As of the latest follow-up on 23 Apr, 2024, the patient reported no discomfort and was living well with an overall survival (OS) of 15 months. The timeline of the patient care was presented in **Figure 3B**.

## Literature review

**Table 1** summarizes seven cases of primary seminal vesicle lymphoma, including five DLBCL and two Burkitt lymphomas. One case is from our own institution, and the remaining six are from the literature. The median age was 58 years. Clinically, patients typically manifest with non-specific symptoms, including abdominal pain, oliguria, and dysuria. However, some individuals may be asymptomatic. The predominant radiological findings include masses or enlargement of the seminal vesicles as observed on transrectal ultrasound and computed tomography or MRI. Four cases demonstrated increased metabolism on PET/CT. The majority of patients with primary seminal vesicle DLBCL received R-CHOP chemotherapy. Definitive OS was documented in two cases, at four and five months, respectively. Another case was monitored for only three months. OS data were not available for three patients. Our patient, with an OS of 15 months at the last follow-up, showed a more favorable outcome.

**Table 1:** Primary seminal vesicle lymphoma in the previous reports and our case.

Investigator	Sex/Age	Lymphoma type	Clinical manifestation	Imaging characteristics	PSA ng/mL	Treatment	Prognosis	OS	Reported
OUYANG J et al. (7)	M/57	BL	Dysuria, hematuresis, inguinal lymphadenectasis	CT: Irregularly low-density shadow in the seminal vesicle	NA	Declined further treatment	NA	NA	2009
ZHU J et al. (8)	M/35	DLBCL	perineal pain	TRUS and CT: large mass in the seminal vesicles	NA	NA	NA	NA	2011
ZHU B et al. (9)	M/63	DLBCL	Lower abdominal pain, oliguria, elevated serum creatinine	MRI: large mass in the seminal vesicles; PET/CT: showing FDG-avid hotspots	2.3	R-CHOP*6	The serum creatinine became normal and the size of the mass decreased.	NA	2012
KWAG K S et al. (10)	M/58	DLBCL	Dysuria, dysfunction	TRUS and CT: large mass in right seminal vesicle; MRI: heterogeneously intermediate-high signal intensity on T2-weighted images; PET/CT: uptake value of 23.5	7.35	R-CHOP	Lesions disappeared; no significant FDG uptake	> 3 Months	2016
GONG W et al. (11)	M/77	DLBCL	Abdominal distension and pain	US and CT: enlargement of the bilateral seminal vesicles; PET/CT: uptake value of 32.3	NA	R-CHOP	Died five months later.	5 Months	2021
WU M et al. (12)	M/49	BL	Dysuria	CT and MRI: enlargement of the seminal vesicle. PET/CT: showed no other tumors.	7.8	Radical surgery	Regained normal urination ability after the surgery. Refused chemotherapy during tumor progression and died.	4 Months	2023
Our case	M/68	DLBCL	Asymptomatic	MRI: Abnormal signal in the left seminal vesicle; PET/CT: uptake value of 6.0 in the fossa of prostate	24.2	R-CHOP*6	CR	>15 Months	2024

BL, Burkitt lymphoma; TRUS, Transrectal ultrasound; US, Ultrasound; CT, Computed tomography; MRI, Magnetic resonance imaging; FDG PET/CT, Fluorodeoxyglucose positron emission tomography-computed tomography; NA, Not applicable; DLBCL, Diffuse large B-cell lymphoma; R-CHOP, rituximab, cyclophosphamide, doxorubicin, vincristine, prednisone; CR, Complete remission; OS, overall survival.

## Discussion

Primary lymphomas of the seminal vesicles are quite rare and DLBCL is the most common type. Symptoms of primary seminal vesicle lymphomas are non-specific and often present with symptoms that mimic those of prostatic diseases. Additionally, patients may experience hemo- and leuco-spermia, perineal discomfort, defecation pain, and complications such as acute kidney injury due to ureteral compression (5, 10). Distinguishing between primary seminal vesicle tumors and secondary spread from adjacent organs like the prostate, bladder, or rectum is critical (14). However, the absence of pathognomonic features on traditional imaging modalities—transrectal ultrasound, computed tomography, and MRI—poses significant diagnostic challenges. The high incidence of prostatic carcinoma invading the seminal vesicles further complicates diagnosis. In this context, PET/CT has emerged as a pivotal tool (15), demonstrating intense FDG avidity in these lymphomas and identifying metabolic changes that precede structural alterations (1, 15). This analysis provides a thorough elaboration of the clinical manifestations, imaging characteristics, diagnosis, treatment modalities and prognosis associated with this rare entity.

In our recent study, we presented a detailed case report of primary DLBCL of the seminal vesicles and conducted a comprehensive review of six additional cases of primary seminal vesicle lymphoma. Remarkably, our case initially diagnosed with prostate cancer was subsequently found to have primary seminal vesicle lymphoma during routine surveillance. The diagnosis of primary seminal vesicle DLBCL in this patient is complex and challenging. Immunohistochemical analysis in our case revealed positivity for CD20, CD10, Bcl-6, CD5, PAX5, C-myc (50%), MUM1, and Ki-67 (80%), with negative staining for CD3 and Bcl-2. Based on immunohistochemical analysis, along with the results of clonal rearrangement and genetic analysis, the diagnosis of DLBCL is confirmed. However, some involvement of the prostate fossa cannot be completely ruled out due to the concurrent presence of prostate cancer. This situation complicates our diagnosis and highlights the potential for misdiagnosis or delayed diagnosis, particularly in cases with preexisting prostate cancer. Such delays can significantly impact prognosis, emphasizing the need for heightened clinical suspicion and definitive pathological confirmation. Consequently, relevant examinations including imaging, pathomorphology, immunohistochemistry, and sequencing must be refined before a definitive diagnosis can be established.

The subtype of MCD characterized by MYD88^L265P^ and/or CD79B^mut^ (16). Our case represents the first reported instance of utilizing next-generation sequencing to identify the MCD subtype of DLBCL in primary seminal vesicle lymphoma. This discovery not only enriches our understanding of site-specific lymphomas but also carries significant clinical implications. The MCD subtype is associated with a high risk of central nervous system (CNS) recurrence and poor prognosis (17). While CNS prophylaxis is advocated, the optimal prevention strategy remains controversial (17, 18). Bruton’s tyrosine kinase (BTK) inhibitors, capable of penetrating the blood-brain barrier, show promise as potential CNS-preventive agents, although supporting evidence is still nascent (17). In younger patients (<60 years) with MCD DLBCL, combining BTK inhibitors like ibrutinib with R-CHOP has demonstrated improved survival (16). However, this benefit was not observed in older patients, likely due to ibrutinib’s added toxicity leading to reduced chemotherapy tolerance (16). Our case uniquely contributes to this discourse. Despite belong to the MCD subtype classification, the patient received only R-CHOP regimen rather than combination therapy with ibrutinib. This decision was predicated on the patient’s advanced age, frail performance status, multiple comorbidities, and poor tolerance to immunochemotherapy. Indeed, severe adverse reactions during the first treatment cycle necessitated dose reductions in subsequent cycles. In addition, the patient did not undergo lumbar puncture and intrathecal chemotherapy because of lumbar spine disease. Due to the patient’s striken in years and the chronic nephritis, high-dose methotrexate chemotherapy was not administered. Nevertheless, the patient achieved complete metabolic remission after six cycles of R-CHOP and remains disease-free at the latest follow-up.

This case illuminates the complexity of tailoring treatment strategies for elderly patients with high-risk DLBCL subtypes. While targeted therapies like ibrutinib show promise in younger populations, their role in older, frailer patients remains uncertain. Our experience suggests that standard R-CHOP can still yield favorable outcomes in this demographic, underscoring the need for further research to optimize treatment paradigms in this challenging subgroup.

## Conclusion

In summary, our study not only elucidates the clinical presentations, pathological features and imaging characteristics of primary seminal vesicle lymphomas but also advances our molecular understanding of these rare tumors. By highlighting diagnostic difficulty, the utility of advanced imaging, and the complexities of personalized treatment in high-risk subtypes, we aim to enhance clinical recognition, facilitate accurate diagnosis, and guide therapeutic decisions, ultimately improving patient outcomes in this under-recognized malignancy.

## Data Availability

The original contributions presented in the study are included in the article/**Supplementary Material**. Further inquiries can be directed to the corresponding author.
