# Supplementary material for: Primary seminal vesicle diffuse large B-cell lymphoma: a case report and review of the literatures
Source: Front Immunol. 2024 Oct 30;15:1461090. doi: 10.3389/fimmu.2024.1461090 (PMC11557305; doi:10.3389/fimmu.2024.1461090)
Supplement: Supplementary file 1 [file DataSheet1.pdf]

CARE Checklist of information to include when writing a case report

CARE case report guidelines

(cc) BY-NC-ND

Topic	Item	Checklist item description	Reported on Line
Title	1	The diagnosis or intervention of primary focus followed by the words "case report"	Line 3
Key Words	2	2 to 5 key words that identify diagnoses or interventions in this case report, including "case report"	Line 63-64
Abstract (no references)	3a	Introduction: What is unique about this case and what does it add to the scientific literature?	Line 57-61
	3b	Main symptoms and/or important clinical findings	Line 49-51
	3c	The main diagnoses, therapeutic interventions, and outcomes	Line 51-56
	3d	Conclusion—What is the main "take-away" lesson(s) from this case?	Line 59-61
Introduction	4	One or two paragraphs summarizing why this case is unique (**may include references**)	Line 80-90
Patient Information	5a	De-identified patient specific information.	Line 93
	5b	Primary concerns and symptoms of the patient.	Line 94-96
	5c	Medical, family, and psycho-social history including relevant genetic information	Line 93
	5d	Relevant past interventions with outcomes	Line 94-95
Clinical Findings	6	Describe significant physical examination (PE) and important clinical findings.	Line 94-95
Timeline	7	Historical and current information from this episode of care organized as a timeline	Line 93-126
Diagnostic Assessment	8a	Diagnostic testing (such as PE, laboratory testing, imaging, surveys).	Line 93-118
	8b	Diagnostic challenges (such as access to testing, financial, or cultural)	Line 101-110
	8c	Diagnosis (including other diagnoses considered)	Line 96-110
	8d	Prognosis (such as staging in oncology) where applicable	Line 121-126
Therapeutic Intervention	9a	Types of therapeutic intervention (such as pharmacologic, surgical, preventive, self-care)	Line 97-98, 120-121
	9b	Administration of therapeutic intervention (such as dosage, strength, duration)	Line 120-123
	9c	Changes in therapeutic intervention (with rationale)	Line 121-123
Follow-up and Outcomes	10a	Clinician and patient-assessed outcomes (if available)	Line 125-126
	10b	Important follow-up diagnostic and other test results	Line 123-125
	10c	Intervention adherence and tolerability (How was this assessed?)	Line 121-123
	10d	Adverse and unanticipated events	Line 121-123
Discussion	11a	A scientific discussion of the strengths AND limitations associated with this case report	Line 147-193
	11b	Discussion of the relevant medical literature **with references**.	Line 147-158, 170-179
	11c	The scientific rationale for any conclusions (including assessment of possible causes)	Line 195-201
	11d	The primary "take-away" lessons of this case report (without references) in a one paragraph conclusion	Line 189-193
Patient Perspective	12	The patient should share their perspective in one to two paragraphs on the treatment(s) they received	Line 120-126
Informed Consent	13	Did the patient give informed consent? Please provide if requested	Yes ☑ No ☐
